# Diagnostics of Thyroid Cancer Using Machine Learning and Metabolomics

**DOI:** 10.3390/metabo14010011

**Published:** 2023-12-22

**Authors:** Alyssa Kuang, Valentina L. Kouznetsova, Santosh Kesari, Igor F. Tsigelny

**Affiliations:** 1 Haas Business School, University of California at Berkeley, Berkeley, CA 94720, USA; alyssakuang@berkeley.edu; 2 San Diego Supercomputer Center, University of California at San Diego, La Jolla, CA 92093, USA; vkouznet@ucsd.edu; 3 BiAna, La Jolla, CA 92038, USA; 4 CureScience Institute, San Diego, CA 92121, USA; 5 Pacific Neuroscience Institute, Santa Monica, CA 90404, USA; santosh.kesari@providence.org; 6 Department of Neurosciences, University of California at San Diego, La Jolla, CA 92093, USA

**Keywords:** thyroid cancer, machine learning, metabolomics, diagnostics

## Abstract

The objective of this research is, with the analysis of existing data of thyroid cancer (TC) metabolites, to develop a machine-learning model that can diagnose TC using metabolite biomarkers. Through data mining, pathway analysis, and machine learning (ML), the model was developed. We identified seven metabolic pathways related to TC: Pyrimidine metabolism, Tyrosine metabolism, Glycine, serine, and threonine metabolism, Pantothenate and CoA biosynthesis, Arginine biosynthesis, Phenylalanine metabolism, and Phenylalanine, tyrosine, and tryptophan biosynthesis. The ML classifications’ accuracies were confirmed through 10-fold cross validation, and the most accurate classification was 87.30%. The metabolic pathways identified in relation to TC and the changes within such pathways can contribute to more pattern recognition for diagnostics of TC patients and assistance with TC screening. With independent testing, the model’s accuracy for other unique TC metabolites was 92.31%. The results also point to a possibility for the development of using ML methods for TC diagnostics and further applications of ML in general cancer-related metabolite analysis.

## 1. Introduction

Thyroid cancer (TC) is the most common endocrine cancer. TC is deadly because of its quickness to spread to the other parts of the human body, such as the kidney, lungs, prostate, and the breasts. While TC is a common cancer, the symptoms typically do not show until the later stages. TC also can recur. Currently, doctors classify thyroid nodules with fine-needle aspiration biopsy (FNAB), a type of evaluation of palpable soft tissue lesions that are clinically suspected to be metastatic carcinomas, which uses an ultrasound (US) image [1]. FNAB is found to be inaccurate 20% of the time [2].

### 1.1. Artificial Intelligence, Computer-Aided Diagnostics, and Machine Learning in Cancer Therapy

Computer-aided diagnostics (CAD) has been used to help radiologists with screening mammography and other cancers. Indeed, in a study conducted on CAD at the University of Michigan, the CAD system recognized 10 of the 11 cancers within the patient cohort [3]. Analysis of thyroid cancer stages can identify the severity of the development of this cancer. Ultrasound (US) is the most widely used diagnostic tool for TC and other cancers. US is enhanced through deep learning (DL), as DL can extract nonlinear features from predictable auditory patterns, which are present in US and are often neither clear nor simple [4]. Artificial intelligence (AI) has been useful in diagnostics of cancer and other diseases. AI is currently used to predict disease outbreaks, increasing the efficiency of operations and quickly navigating large datasets to provide crucial information to doctors. Specifically, AI, as well as CAD, has come to the aid of thyroid cancer diagnostics. Due to current risks and the complicated system of the Thyroid Imaging Reporting and Data System (TI-RADS), CAD systems that are based on AI strategies alleviate the complexity of US-based risk-stratification systems (RSS) and provide higher accuracy for detecting and evaluating potential thyroid nodules [5]. Furthermore, machine-learning (ML) approaches can help predict and identify nodules with a high risk of mutations through molecular testing [6]. ML largely relies on probability and statistics and is inherently more powerful, since it enables conclusions or decisions that would otherwise be impossible to make using traditional statistical methods [7]. In a study conducted by Li et al., authors developed a classification model using groups of over 300,000 images [8]. Several CAD systems based on US features have been created using the ML method to analyze thyroid imaging. There are also several commercial analysis systems for such image recognition. One of these systems, AmCAD-UT, is used for characterizing thyroid nodules through statistical pattern recognition, as well as quantified algorithms [5].

ML can conduct complex tasks, such as introducing a human-like interpretation of an image [9]. ML, and in particular DL, has been incorporated into lung cancer research. AI models have been used to help doctors identify lung cancer on CT scans. Furthermore, applications of ML into US, including image registration and content retrieval, are developing. Cancer is a complex disease with various causes and various outcomes for different people. Therefore, the use of combining clinical data, medical images, various biomarkers, and ML strategies would allow for a more holistic approach to diagnosis and possible prevention. ML is used for both cancer detection and enhanced recognition of cancer progression. For almost 20 years, decision trees and artificial neural networks (ANNs) have been employed in the detection and diagnosis of cancer [10,11,12]. The prognosis of cancer is determined by a variety of clinical criteria, such as the patient’s age and general health, the type and location of the disease, as well as the grade and size of the tumor [13,14,15]. ML takes this data, compares it, and forms predictions that could characterize a certain node and aid in assessing the severity of a current patient’s condition. Indeed, scientists have grown reliant on protein markers and microarray data, which largely apply to breast and prostate cancers [7]. ML utilizes unsupervised and supervised techniques, which are mostly used for classification and regression. Abundant data, AI, and ML algorithms are being utilized more and more frequently in medicine and health for things like disease diagnosis, individualized treatment plans, medication development, clinical trial research, and the prediction of epidemic outbreaks. The metabolic features of lung cancer can be revealed by metabolomics technology [16]. Researchers say that ML helps with diagnostics through analyzing pathology profiles and clinical images and converting data into algorithmic sequences [17]. ML has the potential to analyze and identify specific types of cancer, predict cancer susceptibility, and screen individuals for cancer regularly before it can spread. ML models have also been developed to help doctors conduct CT scans and project images of cancer [9]. DNA strains and variants that indicate early-stage risk factors for cancer and other disorders have been identified with the help of ML [18]. ML methods are the most effective in detecting patterns of changes in multivariate data. In clinical trials that compare various treatment approaches, the resulting ML models could subsequently be utilized to enhance the precision of tumor diagnosis and to select the best treatment plans.

Multilayer perceptron (MLP), recurrent neural network (RNN), and convolution neural networks (CNN) are the most common neural networks that are involved with oncology. Traditionally, histology or cytopathology is used to diagnose cancer. These forms of assessment examine whether tumor cells are present in a given patient’s sample [19]. Histology-based CNNs have proved to be successful at grading cancer in the prostate, breast, and colon [20]. DL models have successfully classified healthy versus cancerous tissues in lung cancer using whole-slide imaging. A challenge is locating the unknown sites where cancer originates from in a body. Around 3–5% of cancer cases are cancers of unknown origin [21]. ML has helped with this greatly. Using transcriptome data and DL approaches, it is possible to forecast the origin of tumors.

### 1.2. Radiomics and AI for Diagnostics of TC

Recently, radiomics has also been a rising method for applying AI in diagnosing potential TC patients. Radiomics is performed through inputting medical images, and AI processes the data and performs deeper data mining through analyzing the image itself compared to other similar images and using specific features extracted to accurately classify and predict models. Radiomics involves image acquisition, image segmentation, feature extraction, and feature selection [22]. For image segmentation, the process can be performed either manually or semi-automatically. Manually extracting features of images and inputting them into a larger system is the typical method. Radiomics eliminates possible biases when interpreting data and allows for the repeatability of interpretations. This repeatability allows AI-based algorithms to discover possible patterns and allows researchers to generalize from abnormal findings. For radiomics, although it is unable to completely replace human operations within radiology, the image interpretation efficiency can be increased using ML methods. However, just like DL, the limitation of radiomics is the large amount of data required to build models and the lack of equipment. Even so, the future of AI continues to grow, with potentials of better assessments when improving the tools necessary and better generalizability.

### 1.3. Metabolomics in Cancer Diagnosis

Metabolomics research and ML open doors for diagnostics of cancer. The metabolic pathways and metabolites that control the growth of tumors and physiological function have been identified [23]. Blood metabolites have been shown to be helpful in non-invasive diagnostic methods for several disorders, including lung malignancies. They can also serve as biomarkers when evaluating a tumor [24]. Monitoring metabolites can help track the stage of the tumor’s progression and even identify responses to drugs and other treatments [25]. A key indicator of cancer is an increase in glucose consumption by tumors. This glucose consumption is monitored by fluorodeoxyglucose positron emission tomography (FDG-PET) imaging [26]. With the glucose increase, the various pathways that facilitate energy production are activated. The pathways that are identified in cancer patients contribute to the prognosis of cancer developing. ML models are able to analyze the imaging of the changes in a tumor’s environment to identify patterns for possible pathways or increases in glucose.

Many publications describe the metabolites involved in different types of cancers. In the exploration of metabolites and biomarkers that could identify stages of bladder cancer (BCa), for example, ML methods were used to compare metabolite patterns at various stages of BCa [27]. Metabolic pathways, similarly, relay correlations in certain metabolites in normal people versus in patients with oral cancer. Kouznetsova et al., using saliva metabolites, created an ML model that can determine whether oral lesions are caused by oral cancer or periodontitis [28]. Furthermore, ML-based classifiers used genomic data to generate a panel of 16 DNA methylation indicators and, using the random forest ML model, classified lung cancer with an accuracy of 86.54% [29]. Murata et al. [30] analyzed the saliva concentration of 260 metabolites for diagnostics of invasive carcinoma (IC) of the breast. A multiple logistic regression model was developed for diagnostics of IC. The authors also created an ML model based on the alternative decision tree classifier, with the accuracy of diagnostics being above 90%. Xie et al. [25] developed an ML model with the best accuracy obtained with Naïve Bayes and KNN classifiers for diagnostics of lung cancer. They used 61 plasma metabolites. Yao et al. [31] developed an ML system for the diagnostics of ovarian cancer using metabolites as biomarkers. The system’s accuracy of diagnostics was more than 85%. Huang et al. [32] used serum metabolites for ML diagnostics of early-stage lung adenocarcinoma with an accuracy of more than 90%.

As AI continues to grow and technology advances, the use of ML will increase the effectiveness of TC diagnostics and serve as a measure to identify cancer at early stages or even before cancer develops.

## 2. Materials and Methods

### 2.1. Approaches Overview

The programs and databases used to analyze the thyroid cancer data included PubChem [33], the Human Metabolome Database (HMDB, v. 5.0) [34], PaDEL-Descriptor v. 2.21 [35], Waikato Environment for Knowledge Analysis (WEKA, v. 3.8.5) [36], and MetaboAnalyst, v. 5.0 [37,38]. The initial steps were to extract TC metabolite data from existing datasets to use as a selection model. The data for the development of the ML model were selected from a study that identified differential metabolites in the serum of papillary thyroid cancer (PTC) patients with positive and negative ion patterns [39]. These metabolites were only selected if the *p*-value ≤ 0.05, and the fold change (FC) ≥ 1.5. The resulting table included 64 unique PTC metabolites. Sixty-four randomly selected metabolites were included in the training set. Using the PaDEL software, v. 2.2.1, the 1444 of 1D and 2D descriptors of the metabolites were calculated [35]. Utilizing information-based filtration methods, attributes that were less significant were filtered to create specific data that were later inputted into the ML algorithm.

### 2.2. PubChem

PubChem is a database that provides access to an expansive platform full of information regarding chemicals [33]. A utilized aspect of the database is the readily stored simplified molecular-input line-entry system (SMILES) that offers a unique branding to each chemical compound. The SMILES of each of the selected 64 metabolites and 64 random metabolites were obtained.

### 2.3. The Human Metabolome Database

HMDB, v. 5.0, stores detailed information on all metabolites in the human body [34]. To gather a comparable sample, randomized HMDB selections of 64 metabolites were obtained as the “random” dataset for comparison with the selected 64 metabolites from the table.

### 2.4. PaDEL-Descriptor

PaDEL-Descriptor, v. 2.21, is a software capable of calculating molecular descriptors and fingerprints; in present time, PaDEL calculates 1875 descriptors, including 1444 1D and 2D and 431 3D descriptors [35]. PaDEL was used to calculate descriptors of the 128 metabolites.

### 2.5. Waikato Environment for Knowledge Analysis

WEKA, v. 3.8.5, is a software developed to build classification and regression models and contains a collection of numerous ML classifiers [36]. In the class label, the 64 selected metabolites from the table were labeled as selected, while the 64 random metabolites were labeled as random. The significant metabolites’ descriptors were selected with the attribute evaluator InfoGainAttributeEval. The algorithm evaluated the worth of an attribute relative to the class and therefore discarded any extra data that would be unnecessary to classify the whole of the dataset, selecting only 53 attributes out of the 1546. This provided the highest accuracy in classification. On the preprocessing step, the unwanted instances were removed through the attribute filter. WEKA was also used for cross-validation, assessing the accuracy in which the model could classify if a metabolite was TC related. In this context, accuracy was defined as the number of metabolites that were accurately categorized as being related to thyroid cancer out of the total amount of metabolites assessed. If all of the metabolites were classified as TC metabolites, that would constitute a 100% accuracy. The reason that accuracy must be assessed is to see the capability of the ML model built. If the ML model can consistently and accurately identify TC-related metabolites, it would be easier to identify TC early on.

### 2.6. MetaboAnalyst

MetaboAnalyst, v. 5.0, is a web-based tool that facilitates metabolomics data analysis [37]. The pathway analysis function within MetaboAnalyst 5.0 was used in this paper to identify which pathways were expressed in the 128-metabolite dataset [38]. This tool aided in finding related pathways and creating a chart to show the identifications. On the resulting graph, both the *p*-value and the pathway impact were relevant. The *p*-value was defined as the probability that the statistical summary would be equal to or more extreme than the actual observed results. The pathway impact was defined as an objective estimate of the importance of a given pathway.

## 3. Results

### 3.1. Metabolite Set

For development of a machine learning system for diagnostics of thyroid cancer, we employed a dataset of metabolite biomarkers elucidated by Du et al. [39] (Table 1).

### 3.2. Metabolic Pathways

**Figure 1:**
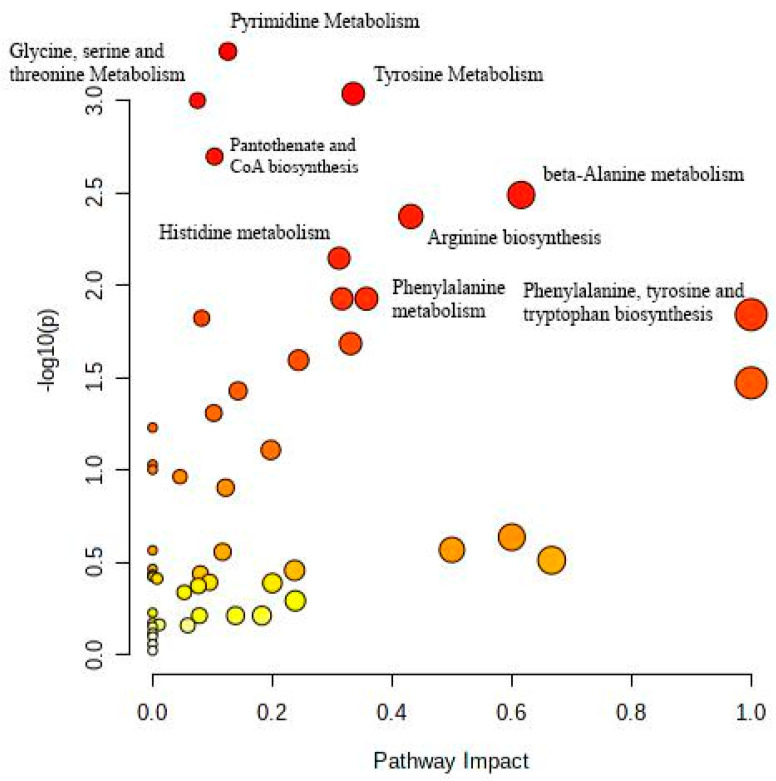
Pathways were matched to the metabolites by MetaboAnalyst, v. 5.0, and presented in a diagram. The position on the y-axis and the vibrancy of the color were determined by the *p*-value; the higher the value on the y-axis and the darker the red color, the greater the significance. Blue dots signified the least significance, while the orange dots signified a high significance just under red. The darker the red, the greater the significance of the pathway. The x-axis and size of a point signified pathway impact values. The most significant pathways were Pyrimidine metabolism, Tyrosine metabolism, and Glycine, serine, and threonine metabolism.

The metabolic pathways described proved that the selected metabolites were related to thyroid cancer or cancer generally (Figure 1).

#### 3.2.1. Pyrimidine Metabolism

Pyrimidine synthesis includes several enzymes that are overexpressed in PTC and thyroid cancer in general. The phosphorylation of uridine monophosphate to uridine diphosphate is overexpressed in PTC [40]. Likewise, in the lungs, where TC can easily spread to, the pyrimidine metabolism signaling pathway is considerably enriched in over 1290 lung carcinoma samples [41].

#### 3.2.2. Tyrosine Metabolism

Tyrosine metabolism is affected in esophageal cancer [42,43]. Upregulation of tyrosine in serum in hepatocellular carcinoma (HCC) was reported by Watanabe et al. [44]. Systematic examination of the tyrosine metabolic pathway described several genes that participated in this pathway affected in HCC [45]. Alteration of tyrosine metabolism was shown for thyroid cancer [46].

#### 3.2.3. Glycine, Serine, and Threonine Metabolism

The expression of Glycine, serine, and threonine metabolism-related proteins vary among different subsets of TC, with more expression in PTC. In a study investigating the Glycine, serine, and threonine metabolism related to different thyroid cancers, Sun and co-authors found that the use of targeted therapy against the Glycine, serine and threonine metabolism pathway’s numerous sites held potential for TC therapeutic approaches [47]. Furthermore, Glycine, serine and Threonine metabolism-related proteins and enzymes were associated with thyroid tumors.

#### 3.2.4. Pantothenate and CoA Biosynthesis

Decreased levels of CoA-SH were found in both cancerous and normal tissues of animals that had malignant tumors. Furthermore, participating in the biosynthesis of CoA genes, noncoding RNAs can cause cancer [48]. Another study that tested groups of mice with tumors and measured CoA levels corroborated the association of decreased levels of CoA with the potential for cancer and other diseases, and it was found that the growth of the TLX-5 lymphoma was in association with “significant decreases in ‘total’ CoA, and CoA-SH” [49].

#### 3.2.5. Arginine Biosynthesis

Studies on cancer cells with low levels of ASS1 that were denied arginine contributed to the current understanding of the impact of arginine on cancer metabolism [50]. Transcription suppression of metabolic genes within processes such as glycolysis, purine, and pyrimidine synthesis, DNA repair genes, oxidative phosphorylation (OXPHOS), and mitochondrial activities is brought on by arginine deprivation [51]. Furthermore, mitochondria are the target of arginine deprivation. This is harmful, since arginine is a crucial regulator of actions in cancer metabolism in the mitochondria. Tumor cells adapt to stay present by altering metabolic pathways. The reduced inherent capability of tumor cells to create arginine is one of the most prevalent metabolic abnormalities [52].

#### 3.2.6. Phenylalanine Metabolism

Phenylalanine metabolism involves the conversion of phenylalanine into tyrosine and then to acetoacetic acid and fumaric acid. Phenylalanine concentration in the blood is elevated due to Phenylalanine metabolism [53]. Increased blood concentrations of phenylalanine are typical in patients with HIV infection and cancer; however, the exact cause of this phenomenon is yet undiscovered. Phenylalanine metabolism has been found to be one of the main pathways involved in PTC development, the most commonly identified form of thyroid cancer [39]. Generally, for cancer, another study by Abasov found that phenylalanine was present in 80% of cancer cases, as opposed to 56% in the study’s control [43,54].

#### 3.2.7. Phenylalanine, Tyrosine, and Tryptophan Biosynthesis

In a study investigating and identifying certain metabolic characteristics through blood samples in PTC patients, the comprehension of the biological and pathological components involved in the transition from a normal to eventually malignant state can be improved by the identification of specific metabolites exhibiting altered levels of their associated metabolic pathways. Such altered pathways include changes in Phenylalanine, tyrosine, and tryptophan biosynthesis [55].

### 3.3. Machine-Learning Classifiers

**Figure 2:**
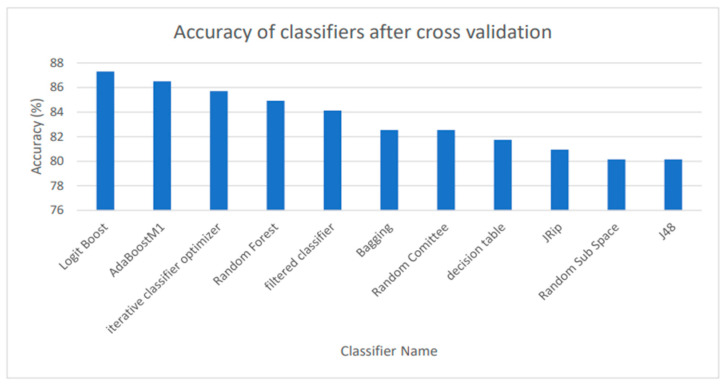
Using cross-validation, the highest classifier performances are shown. The accuracy of cross-validation is the percentage of the metabolites that is deemed relevant to the model through each classifier.

Machine-learning has had rising popularity in both cancer studies and identification of biomedical patterns in data. The final dataset contained 128 metabolites, 64 selected from a previous study and 64 randomly selected from a database of metabolites. The filtered dataset had a total of 53 attributes, including fold change (FC). We tried multitudes of classifiers, including Naïve Bayes, MultilayerPerceptron (MLP), AttributeSelectedClassifier, and more. The highest accuracies of classification were with the classifiers LogitBoost, AdaBoostM1, iterative classifier optimizer, RandomForest, filtered classifier, Bagging, Random Committee, decision table, JRip, Random Sub Space, and J48, all with accuracies above 80%, with the highest being LogitBoost with an 87.03% accuracy (Figure 2). The classifiers were carried out through ten-fold cross-validation, the function that takes the dataset, divides it into ten pieces or folds, and tests each piece on the remaining nine pieces for training (as an estimate of the model), testing each distinct piece 10 times. Then, WEKA averaged the results for each test to obtain the accuracy of each classifier. Cross-validation reduced the variance of the estimate.

### 3.4. Receiver Operating Characteristics

**Figure 3:**
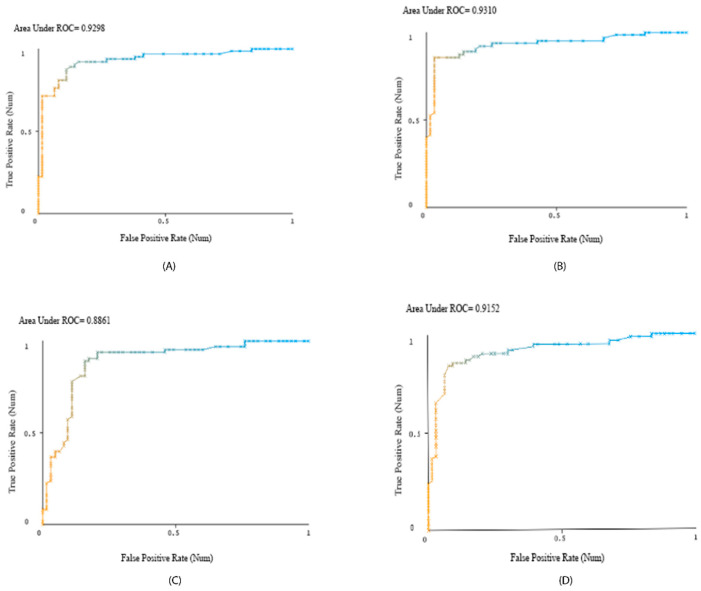
Through WEKA, ROC curves were generated for the highest accuracy classifiers with respect to the dataset: LogitBoost (**A**), AdaBoostM1 (**B**), Random Forest (**C**), and iterative classifier optimizer (**D**). Position on the y-axis signifies a classifier’s positive response rate when the correct response was positive, and the x-axis signifies a classifier’s positive response rate when the correct response was negative.

**Figure 4:**
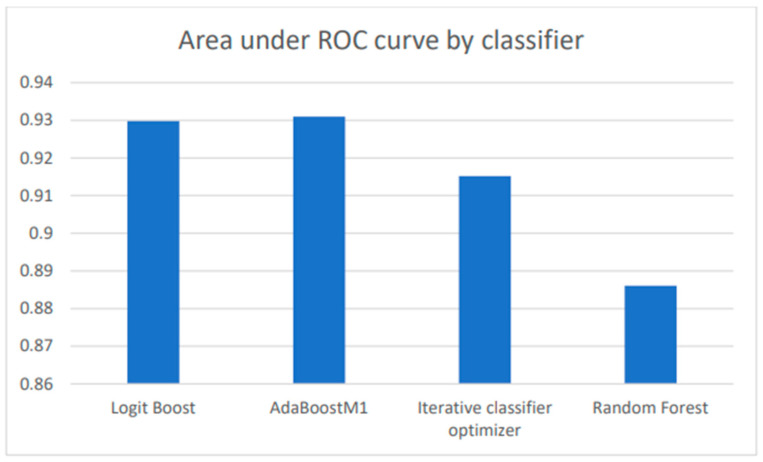
The areas under the ROC curves (AUC) for the highest accuracy classifiers are shown. The AUC is the overall measure of performance in each classifier.

**Figure 5:**
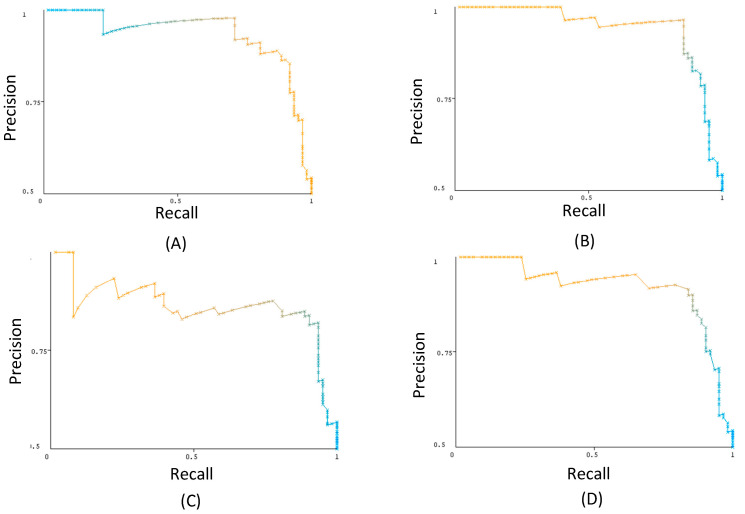
Precision–recall curves were generated for the highest accuracy classifiers in respect to the dataset: LogitBoost (**A**), AdaBoostM1 (**B**), Random Forest (**C**), and iterative classifier optimizer (**D**).

The receiver operating characteristic (ROC) curve shows a classifier’s accuracy and diagnostic power at all class threshold levels. Both the true-positive rate and the false-positive rate are presented by the model. The area under the ROC curve (AUC) is the integration of the AUC from 0 to 1, examining how well predictions are produced at various decision thresholds. The ROC curves with AUCs for top classifiers are presented in Figure 3, and comparisons of their AUCs are presented in Figure 4. The precision–recall curves are presented in Figure 5. These curves confirm that all presented classifiers are solid classifiers.

### 3.5. Independent Testing

In order to test a model to identify potentially TC-related metabolites, an unrelated metabolite set must also be tested, so 34 unique kidney disease metabolites, unrelated to TC, were obtained. These kidney disease metabolites were tested on the developed ML models. After setting the new kidney disease descriptors set as the supplied test, we re-evaluated the kidney disease descriptors onto the TC RandomForest model. The resulting classification of 7% signified that the ML model classified these metabolites as mostly random metabolites.

Similarly, TC metabolites that did not include any of the compounds used for training from the original set that the model was developed from were selected to test the ML model. This was performed to determine whether the model could accurately and consistently identify TC metabolites in screening. A set of 13 TC serum metabolites were selected [56] (Table 2). After conducting a re-evaluation on the same LogitBoost training set model, we found that the ML model classified all metabolites into TC metabolites. The accuracy of the test was 100%. With such a percentage, the ML model was able to accurately assess related metabolites.

## 4. Discussion

Although ML has been significantly developed and improved in past decades, it is interesting to explore whether ML could be used for clinical medical diagnosis. Obermeyer et al. revealed that ML application in clinical laboratory data could drastically improve both prognosis and diagnostic accuracies [57]. Previously, research suggested that ML models have the potential to diagnose diseases such as bladder cancer [58]. It is important to use the results to analyze the applications of AI and ML on thyroid cancer diagnostics. In this study, the main models and findings were developed from previous PTC patient’s’ data. We prepared supplemental material including the Figure S1 that in simplified form explains the concept of model creation using metabolites descriptors. We aimed to set up a model that could assess TC-related metabolites. Therefore, the ML model is a testament to developing future models and not recommended to be directly applied to diagnostics of TC.

After data mining, we used cross-validation in order to counter overfitting or failing to accurately generalize a pattern. We used WEKA to assess accuracy, which is defined by the number of metabolites that are accurately categorized as TC-related out of the total amount of metabolites assessed. Assessing the accuracy helps to understand how capable the model is and, consequently, its ability to identify TC early on. Data mining is crucial to ML, due to the complex nature of ML in big data [59]. The highest scoring classifier, LogitBoost, produced a classification of 87.30%. In Chen and Pan’s study of diabetes, LogitBoost was a highly successful classifier for the machine-learning model using clinical test results [60]. The accuracy of 87.3% meant that in the dataset that contained all thyroid cancer metabolites, the classifier was able to accurately identify a large majority of TC-containing metabolites. Furthermore, the model used to perform independent testing was produced through another relatively accurate classifier, RandomForest. In order to ensure that the model would not falsely classify TC-irrelevant metabolites, we utilized independent testing. The goal of independent testing was to input TC-related datasets and TC-unrelated datasets to see if the model would correctly or incorrectly classify attributes to TC. In response to kidney disease metabolites, the model only classified 7% of the metabolites as TC-related. The ML model largely classified the kidney disease metabolites as random. Furthermore, in response to a related set of TC metabolites, the model was able to identify 92.31% of the data as TC. This established that the model was able to detect relevant TC metabolites and filter TC-irrelevant metabolites. This model could be applied to future uses of AI models in diagnostics for TC. The metabolite dataset was analyzed with MetaboAnalyst and elucidated the most significant pathways and among them TC and cancer in general. MetaboAnalyst generated a graph that identified various pathways related to the dataset and portrayed the most significant pathway impacts. The seven identified pathways included: Pyrimidine metabolism, Tyrosine metabolism, Glycine, serine, and threonine metabolism, Pantothenate, and CoA biosynthesis, Arginine biosynthesis, Phenylalanine metabolism, and Phenylalanine, tyrosine, and tryptophan biosynthesis. These pathways are a sign of abnormal TC and general cancer metabolism.

Through data mining, pathway analysis, and independent testing, a model was developed for PTC patients. As more data were collected and accessed, similar models could be developed with increased accuracies.

### Limitations

Although there have been successes in diagnosing TC patients through AI-generated models, there could be more efficient and accurate ways to diagnose future patients. In order to have more accurate models built, relevant data that is available should be stored in one general location that is easily accessible for research. ML models such as the one created in this paper could be applied and remodeled using more existing data for accurate diagnostics.

## 5. Conclusions

Metabolomic analysis evaluation on PTC serum proved effective in identifying potential thyroid patients through screening. Metabolomics, although not used consistently for screening, has the potential to identify certain molecular markers, which could classify pathological conditions and create higher accuracies within diagnostics. The future of diagnostics through machine learning has exponential potential that could change the entire field of cancer research and cancer diagnostics.

Within the model developed in this project, an accurate machine-learning diagnosis classifier of thyroid cancer was developed. The model can diagnose thyroid cancer based on serum metabolites. Improvements to the developed model can be achieved by adding more metabolites. Further, the model can also be expanded to include different types of thyroid cancer, as the current model is based only on PTC-related metabolites. As the use of ML increases, TC and other cancers alike can be diagnosed with improved efficiency.

The implication of this study is that ML based on metabolite biomarkers is an efficient tool to diagnose TC patients. With the increase in using ML with existing and new biomarkers, the process of diagnosis will greatly improve.

## 6. Future Work

We plan to use a deep-learning strategy for PTC diagnostics using metabolite biomarkers. We also plan to expand the studies toward including, to the ML model the other biomarkers, especially images. It will significantly improve the general diagnostics of PTC.

## Figures and Tables

**Table 1:** The training dataset was derived from the following set of 64 metabolites [39].

Metabolite Name	FC	log(FC)
Quillaic Acid 3-[galactosyl-(1->2)-glucuronide]	0.33217	−1.59000
Quillaic acid 3-[xylosyl-(1->3)-[galactosyl-(1->2)]-glucuronide]	0.32061	−1.64110
L-Aspartyl-L-phenylalanine	0.27591	−1.85770
L-Histidine	0.40090	−1.31870
Pyridinoline	0.40468	−1.30510
5-hydroxylysine	0.47622	−1.07003
L-glutamic acid	0.51228	−0.96499
Hydrogencarbonate	0.58709	−0.76835
L-phenylalanine	2.09010	1.06360
Trans,trans-muconic acid	0.53257	−0.90896
Taurocholic acid	2.21440	1.14690
Disulfiram	0.61107	−0.71060
Citric acid	0.54732	−0.86954
Dimercaprol	0.52417	−0.93190
Argininic acid	0.57260	−0.80439
Ursocholic acid	1.84740	0.88546
Methylmalonic acid	0.42889	−1.22130
Nicotine glucuronide	2.22150	1.15150
L-Kynurenine	0.46308	−1.11070
2-Hydroxyethinylestradiol	2.97520	1.57300
Oleoylcarnitine	2.03350	1.02400
Retinyl beta-glucuronide	2.06240	1.04430
*N*-Acetyl-L-arginine	0.53088	−0.91355
Cyanate	1.86650	0.90033
10-Hydroxy-octadec-12Z-enoate-9-beta-D-glucuronide	2.06310	1.04480
Biotin	0.60751	−0.71902
2-Arachidonylglycerol	0.55461	−0.85046
Beta-alanine	0.55975	−0.83715
3-Indoleacetic acid	0.48593	−1.04120
Acitretin	2.69310	1.42930
Hippuric acid	0.55416	−0.85163
L-Tryptophan	0.61765	−0.69513
Ribothymidine	0.49838	−1.00470
3-Hydroxy-cis-5-tetradecenoylcarnitine	0.61797	−0.69440
*N*-Acetylornithine	0.39628	−1.33540
Threonic acid	1.92510	0.94496
Oxalic acid	0.50332	−0.99047
Alpha-tocotrienol	1.98070	0.98599
Acetone	0.54805	−0.86763
4’-*O*-Methylcatechin	1.67120	0.74089
Glucosylgalactosylhydroxylysine	1.63340	0.70790
L-Tyrosine	0.65647	−0.60719
1-Methylguanosine	0.60407	−0.72721
Azelaic acid	0.36788	−1.44270
4-hydroxybenzaldehyde	0.47227	−1.08230
(S)-3,4-Dihydroxybutyric acid	0.62090	−0.68756
Heparan sulfate	1.64210	0.71556
4-glutathionyl cyclophosphamide	0.62610	−0.67553
Uric acid	0.54884	−0.86554
Thiamine pyrophosphate	1.80870	0.85496
Phenylalanylphenylalanine	0.60089	−0.73483
Farnesyl pyrophosphate	0.55865	−0.83999
p-Cresol sulfate	0.44465	−1.16930
3-hydroxyhexadecadienoylcarnitine	1.87230	0.90484
Hesperetin 3’,*7*-*O*-diglucuronide	1.61650	0.69291
Glucose 6-phosphate	0.54630	−0.87223
Proline betaine	3.83720	1.94010
Dopamine	0.66314	−0.59262
3’-hydroxy-e,e-caroten-3-one	1.52140	0.60537
8-hydroxy-deoxyguanosine	0.63158	−0.66296
12(13)ep-9-KODE	1.68180	0.75004
8-isoprostane	0.58342	−0.77739
Maltotetraose	0.52038	−0.94235
Oxypurinol	0.54446	−0.87710

*Note*: Selected from the dataset of metabolites from an open source [39].

**Table 2:** The independent testing dataset was derived from the following 13 metabolites [56].

Metabolite	
Lauric acid propyl ester	2-monostearin
Pentadeconic acid, glycerine-(1)-monoester	Decanoic acid decylester
Ricinoleic acid	Myo-inositol phosphate
Heptadecanoic acid, glycerine-(1)-monoester	D-gluconic acid
Nonadecanoic acid-glycerine-(1)-monoester	Succinic acid
Eicosanoic acid propyl ester	Citric acid
Hexadecanoic acid propyl ester	

## Data Availability

All data generated for this study are included in the article; further inquiries can be directed to the corresponding author. The data are not publicly available due to their availability from the other studies [22].

## Supplementary Materials

The following supporting information can be downloaded at: https://www.mdpi.com/article/10.3390/metabo14010011/s1, Figure S1: Simplified scheme of ML system function.

## Abbreviations

AI, artificial intelligence; BCa, bladder cancer; CAD, computer-aided diagnostics; FNAB, with fine-needle aspiration biopsy; HCC, hepatocellular carcinoma; HMDB, Human Metabolome Database; TC, thyroid cancer; FC, fold change; InfoGain, information gain; ML, machine learning; PTC, papillary thyroid cancer; PCA, principal component analysis; ROC, receiver operating characteristic; SMILES, simplified molecular-input line-entry system; TIRAD, Thyroid Imaging Reporting and Data System; US, ultrasound; WEKA, Waikato Environment for Knowledge Analysis.
