# Supplementary material for: Diagnostics of Thyroid Cancer Using Machine Learning and Metabolomics
Source: Metabolites. 2023 Dec 22;14(1):11. doi: 10.3390/metabo14010011 (PMC10818630; doi:10.3390/metabo14010011)
Supplement: Supplementary file 1 [file metabolites-14-00011-s001.zip › metabolites-2714032-supplementary.pdf]

metabolites

MDPI

Article

Diagnostics of Thyroid Cancer Using Machine Learning and Metabolomics

Alyssa Kuang [1], Valentina L. Kouznetsova [2,3,4], Santosh Kesari [5] and Igor F. Tsigelny [2,3,4,6,*]

1 Haas Business School, University of California at Berkeley, Berkeley, CA 94720, USA; alyssakuang@berkeley.edu
2 San Diego Supercomputer Center, University of California at San Diego, La Jolla, CA 92093, USA; vkouznet@ucsd.edu
3 BiAna, La Jolla, CA 92038, USA
4 CureScience Institute, San Diego, CA 92121, USA
5 Pacific Neuroscience Institute, Santa Monica, CA 90404, USA; santosh.kesari@providence.org
6 Department of Neurosciences, University of California at San Diego, La Jolla, CA 92093, USA
* Correspondence: itsigeln@ucsd.edu

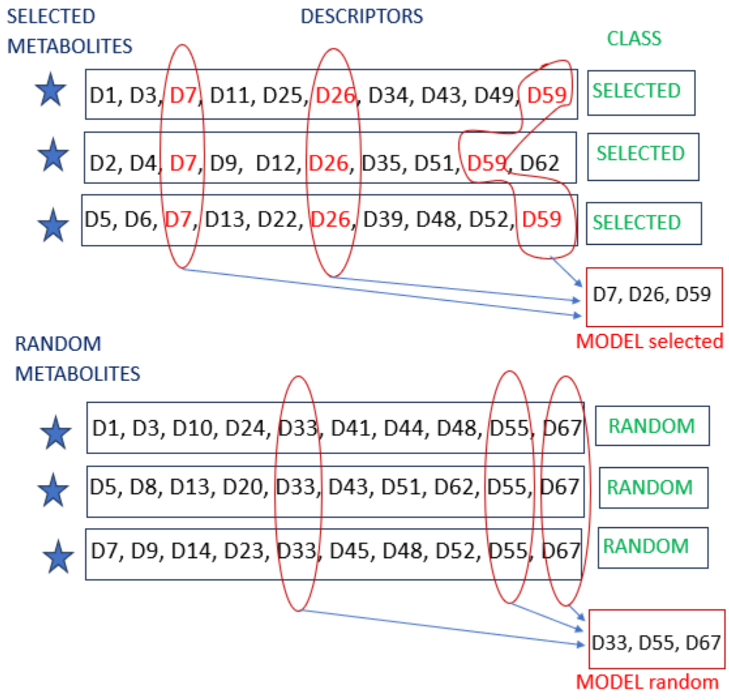

Figure S1. Simplified scheme of ML system function.

Simplified scheme of ML system function. Properties of metabolites corresponding to a specified disease are presented as a set of descriptors related to their 2D and 3D structures. Currently more than 3000 descriptors are invented (for example number of N atoms, or charge). When a set of disease-related metabolites is analyzed with machine learning (ML) system it selects the common descriptors for the metabolites of the set and build based on them the ML model. To select the descriptors for the model ML system uses various classifiers (for example, well known Random Forest). The real scheme of selection and building of the MODEL are much more sophisticated, nevertheless the scheme gives the understanding that NOT THE NAMES OR CHEMICAL STRUCTURES OF metabolites are used in ML system, but the summary of their common descriptors. That is why the resulting MODEL is UNIVERSAL. It can be allied to ANY set of metabolites not even containing a single metabolite from the training dataset. There can be completely new metabolites that were just discovered!
